# Neogenin May Functionally Substitute for Dcc in Chicken

**DOI:** 10.1371/journal.pone.0022072

**Published:** 2011-07-11

**Authors:** Keith Dai Phan, Louis-Philippe Croteau, Joseph Wai Keung Kam, Artur Kania, Jean-François Cloutier, Samantha Joanna Butler

**Affiliations:** 1 Department of Biological Sciences, University of Southern California, Los Angeles, California, United States of America; 2 Institut de Recherches Cliniques de Montréal, Montréal, Québec, Canada; 3 Montreal Neurological Institute, McGill University, Montréal, Québec, Canada; 4 Department of Neurology and Neurosurgery, McGill University, Montréal, Québec, Canada; 5 Faculté de Médecine, Université de Montréal, Montréal, Québec, Canada; 6 Departments of Anatomy and Cell Biology and Biology, McGill University, Montréal, Québec, Canada; Tokyo Medical and Dental University, Japan

## Abstract

Dcc is the key receptor that mediates attractive responses of axonal growth cones to netrins, a family of axon guidance cues used throughout evolution. However, a *Dcc* homolog has not yet been identified in the chicken genome, raising the possibility that Dcc is not present in avians. Here we show that the closely related family member neogenin may functionally substitute for Dcc in the developing chicken spinal cord. The expression pattern of chicken neogenin in the developing spinal cord is a composite of the distribution patterns of both rodent Dcc and neogenin. Moreover, whereas the loss of mouse neogenin has no effect on the trajectory of commissural axons, removing chicken neogenin by RNA interference results in a phenotype similar to the functional inactivation of Dcc in mouse. Taken together, these data suggest that the chick neogenin is functionally equivalent to rodent Dcc.

## Introduction

The chicken genome is significantly more compact than mammalian genomes [1]. During the 310 million years that chickens diverged from a common ancestor with mammals [2], the chicken genome has achieved a size of 1.05 Gb (Ensembl database, www.ensembl.org). In comparison, the human genome is currently thought to be 3.28 Gb whereas the mouse genome is 3.42 Gb (Ensembl). Moreover, the total number of known or novel protein coding genes identified to date in the chicken genome is significantly smaller than the number of equivalent genes in mammalian genomes. There are estimated to be 16,680 such genes in the chicken genome compared to 21,550 genes in human and 22,670 genes in mouse genomes (Ensembl estimates). It is thus perhaps not surprising that this threefold difference in size between the chicken and mammalian genomes has resulted in a number of genes, and even gene families, being apparently missing from the chicken genome. For example, the International Chicken Genome Sequencing Consortium previously identified that genes encoding the vomeronasal receptors, casein milk proteins and salivary-associated proteins seem to be absent from the chicken genome as well as proteins containing the SCAN domain, a dimerization motif often found in zinc-finger transcription factors [1], [3].

**Figure 1:**
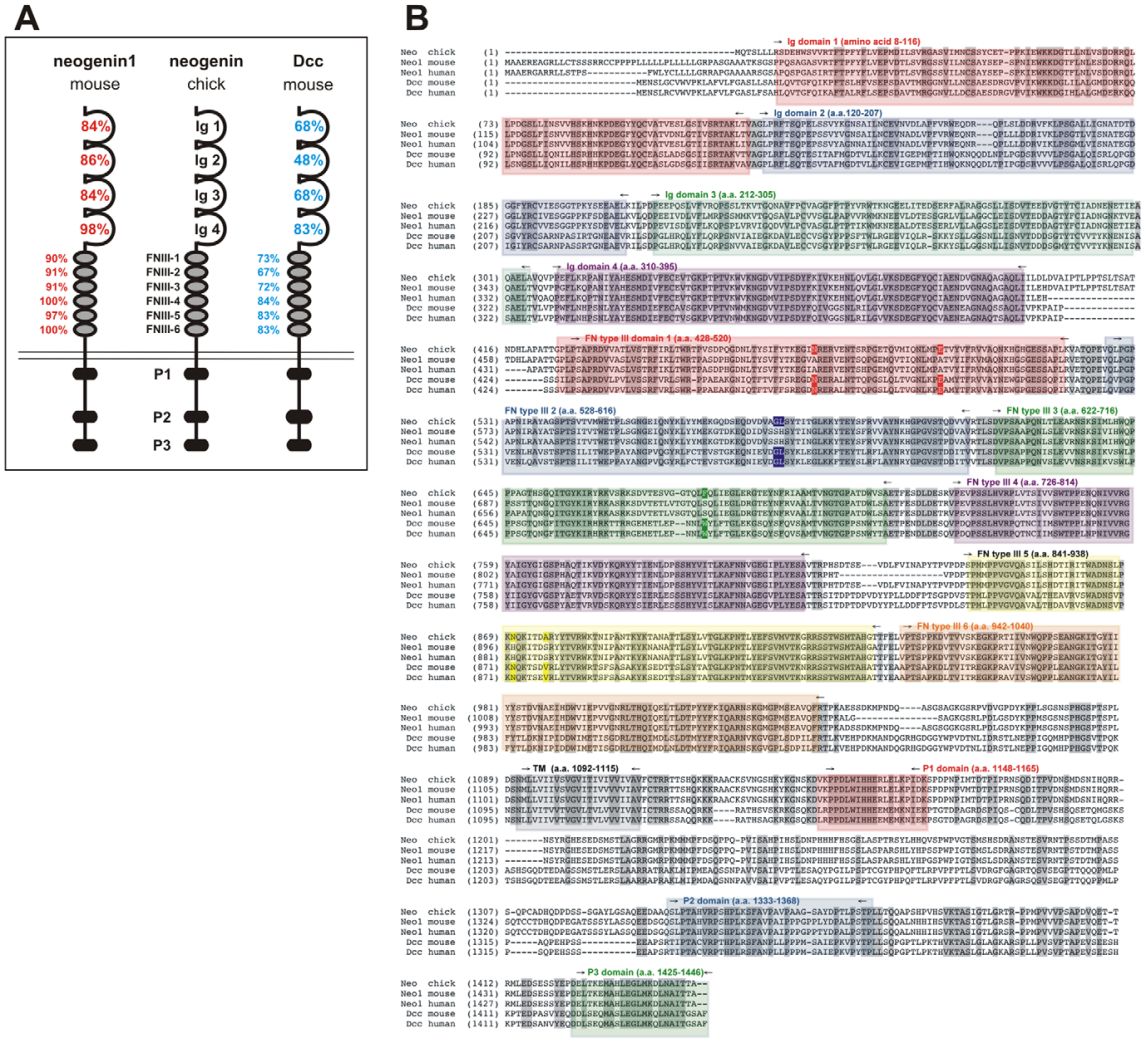
Summary of structure and sequence similarities between chicken, mouse and human neogenin and Dcc proteins. (A) Chicken neogenin is structurally similar to both mouse neogenin1 and Dcc, containing four immunoglobulin (Ig) domains and six fibronectin (FN) type III repeats on the extracellular side and three intracellular P domains. The individual domains of chicken neogenin show highest similarity at the amino acid level to mouse neogenin, although there is also considerable similarity between chicken neogenin and mouse Dcc. (B) Amino acid alignment of chicken, mouse and human neogenin with mouse and human Dcc. Identical amino acids are highlighted in grey. Additionally highlighted in FN domains 1 (red), 2 (blue), 3 (green) and 5 (yellow) are non-conservative amino acid changes that result in chicken neogenin being more similar to human/mouse Dcc than human/mouse neogenin1.

The absence of these genes suggests that other structurally related proteins must be co-opted to functionally substitute for the “missing” genes. Here, we present evidence for such a substitution, chicken neogenin may function in place of the Deleted in Colorectal Cancer (Dcc) protein, since the *Dcc* gene does not appear to be present in the chicken genome. Dcc and neogenin are structurally very similar proteins. They are both members of the immunoglobulin (Ig) domain family with the same secondary structure. They contain four Ig and six fibronectin (FN) type III domains that mediate extracellular interactions, including the specificity of ligand binding [4], [5], [6], as well as three highly conserved P domains critical for intracellular signaling (Fig. 1A, [7], [8]). Dcc was first identified as a tumour suppressor gene [9] but has subsequently been shown to function as a receptor for the highly evolutionarily conserved netrin family of axon guidance signals [10]. The role of Dcc in vertebrate axon guidance was first shown using dorsal commissural axons in the developing spinal cord as a model system. Lhx2/9^+^ commissural neurons [11] are born flanking the dorsal midline and project axons, identified by the presence of the glycoprotein Tag1 [12], ventrally around the circumference of the spinal cord [13], [14], [15]. Dcc is required for commissural growth cones to detect floor plate (FP)-derived netrin1 as a chemoattractant and thereby extend towards the ventral midline [16], [17]. Subsequently, Dcc has been shown to mediate the attractive response of cells towards netrins throughout the nervous system [18], [19], [20]. However, despite the critical role of Dcc in axonal circuit formation, no homolog for Dcc has been identified in chicken despite several efforts to identify it [18], [21].

The role of neogenin has been less extensively studied than Dcc. It is more widely expressed than Dcc [22], [23] and studies have implicated neogenin as having functions throughout development in roles as diverse as cellular survival, cellular adhesion/migration, tissue morphogenesis and differentiation [24], [25]. Recent studies examining the role of neogenin as an axon guidance receptor have focused on its ability to bind repulsive guidance molecule (RGM), a glycosylphosphatidylinostol (GPI)-linked glycoprotein [26] and thereby mediate chemorepulsion [27]. Nonetheless, neogenin has been shown to bind to different netrins [16], [28] and can function to attract axons in the *Xenopus* forebrain towards a source of netrin [29]. Here, we show that chick neogenin has a distribution that appears to be the composite of the expression patterns of rodent Dcc and neogenin. Moreover, the effect of reducing neogenin function in chicken phenocopies loss of function mutations in mouse *Dcc*, but not mouse *neogenin*. Taken together, these results suggest that neogenin has assumed the role of Dcc in chicken.

## Results

### The chicken genome contains a single *neogenin*-like gene

Using the Basic Local Alignment Search Tool (BLAST, http://blast.ncbi.nlm.nih.gov/Blast.cgi) we compared the predicted protein sequence of mouse (*Mus musculus*) Dcc or neogenin splice isoform 1 [30] against the predicted proteome of various species: *Gallus gallus* (chicken), *Danio reno* (zebrafish), *Xenopus laevis*, *Xenopus tropicalis*, *Drosophila melanogaster*, *Caenorhabditis elegans*. This approach confirmed previous findings that the zebrafish [31], [32] and the combined *Xenopus* [33], [34] proteomes contain specific homologs to both mouse Dcc and neogenin1, whereas the *Drosophila* and *C.elegans* proteomes contain only a single homolog called frazzled [7] or Unc-40 [35] respectively. This approach additionally revealed that the chicken proteome contains only a single protein, neogenin, closely related to the protein sequence of both mouse Dcc or neogenin [22].

To further assess the identity of this protein, we used BLAST to compare predicted protein sequences of Dcc and neogenin from the various species to each other. The results are shown in Table 1. In summary, the extent of similarity is high (80–95%) between the vertebrate Dcc or neogenin orthologs and is slightly lower (around 70%) when comparing the vertebrate Dcc and neogenin homologs. In contrast, the invertebrate homologs, frazzled and Unc-40, are equivalently similar (about 50%) either to each other or the vertebrate forms of Dcc or neogenin. Using these comparisons as a guideline, chicken neogenin is clearly most similar to the neogenin orthologs, rather than Dcc orthologs, suggesting that the chicken neogenin is indeed a neogenin ortholog, rather than the hybrid neogenin/Dcc protein seen in invertebrates.

**Table 1:** Comparison of extent of amino acid similarity between Dcc and neogenin in different species.

		Mouse		Chick	Zebra fish		*X. laevis*	*X. tropicalis*	*D. melanogaster*	*C. elegans*
		Dcc	Neo1	Neo	Dcc	Neo	Dcc	Neo	FrazzledA	Unc-40
Mouse	Dcc	100%	70%	70%	83%	69%	90%	71%	57%	53%
	Neo1		100%	94%	67%	82%	70%	87%	51%	52%
Chick	Neo			100%	69%	82%	70%	85%	51%	52%
Zebra fish	Dcc				100%	68%	84%	70%	49%	52%
	Neo					100%	69%	81%	52%	51%
*X. Laevis*	Dcc						100%	73%	50%	50%
*X. Tropicalis*	Neo							100%	58%	52%
*D. Melanogaster*	FrazzledA								100%	51%
*C.elegans*	Unc-40									100%

These observations suggest that the chicken genome does not contain the *Dcc* gene. The chicken genome has been sequenced to 6× coverage, with 95% of the 1.05 Gb genome assigned to chromosomes ([1], http://www.ensembl.org/Gallus_gallus). However, gaps remain in the sequence, thus our failure to identify the chicken *Dcc* gene could rather result from the chicken genome being incompletely sequenced. To assess this possibility further, we examined where the genes flanking *Dcc* in the mouse genome are located in the chicken genome. The mouse *Dcc* gene maps to 71.41–72.51 Mb on chromosome 18 (NCBI, mouse genome, Build 37.2). Interestingly, three genes immediately distal to the mouse *Dcc* gene (*Acca2*, *Rpl17* and *Smad7*, 74.93–75.56 Mb) map to the proximal tip of chicken chromosome Z (0.86–1.13 Mb, NCBI, chicken genome, Build 2.1) whereas genes immediately proximal to the mouse *Dcc* gene (*Mc2r*, *Mc5r* and *Tubb6*, 68.59–67.55 Mb) map to chicken chromosome 2 (98.86–99.60 Mb). Thus, based on the synteny of the mouse genome, the region either side of the putative chicken *Dcc* gene appears to have been separated by a translocation event, making it feasible that the *Dcc* gene was lost. Nonetheless, we cannot rule out the possibility that the chicken *Dcc* gene has not been sequenced yet.

Further analysis of chicken neogenin protein shows that it is most highly similar to mouse neogenin1 at the amino acid level (94%, Table 1, Figure 1B). However, there is considerable sequence similarity between the distinct domains shared between chick neogenin and mouse Dcc (Fig. 1). In particular, the Fibronectin (FN) type III domains generally show over 80% similarity with Dcc. Moreover, there are a few non-conservative changes in amino acid residues in FN domain 1–3, 5 resulting in chicken neogenin being more similar to mouse/human Dcc than mouse/human neogenin1 (Fig. 1B).

Taken together, these observations raise the possibility that *Dcc* is not present in the chicken genome. To assess whether chicken neogenin has an activity that compensates for the loss of Dcc, we have examined the role of neogenin in the developing spinal cord where the function of Dcc has been well described [10], [36].

### The expression pattern of chicken *neogenin* is similar to the patterns of mouse *Dcc* and *neogenin*

**Figure 2:**
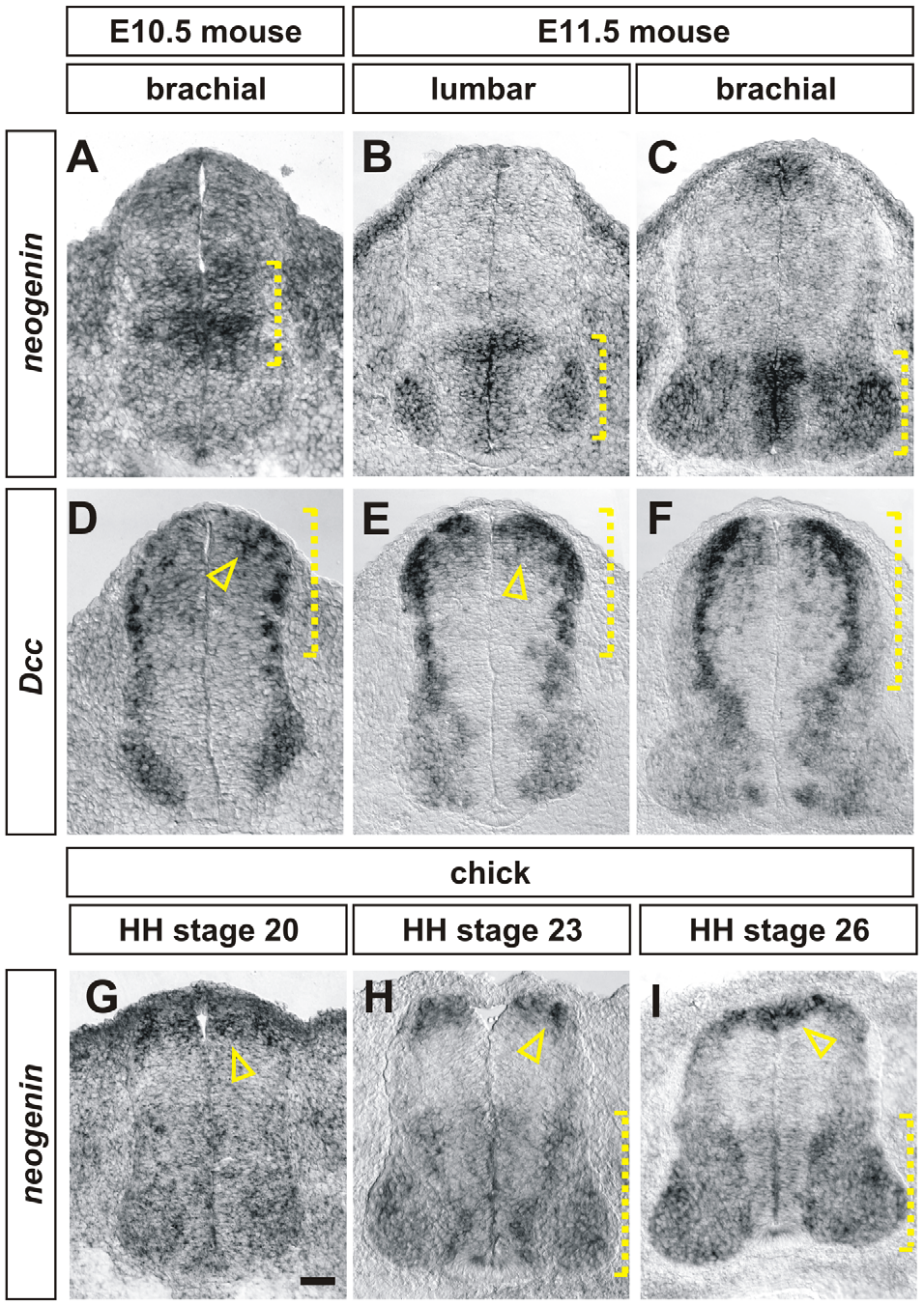
Chick *neogenin* is expressed in a similar manner to mouse *Dcc*. (A–H) *In situ* hybridization experiments for *neogenin* (A–C, G–I) and *Dcc* (D–F) on transverse sections of the spinal cord of E10.5 (A, D) and E11.5 (B, C, E, F) mouse embryos and HH stage 20 (G), 23 (E) and 26 (I) chicken embryos. (A–C) In mouse, *neogenin* is expressed first in the intermediate spinal cord (bracket, A). By E11.5, neogenin is present at high levels in the ventral ventricular zone as well as broadly in motor neurons (brackets, B, C) (D–F) Mouse *Dcc* is expressed at highest levels in the dorsal-most neural progenitors (arrowhead, D, E) and in post-mitotic neurons (brackets, D–F) throughout the dorsal spinal cord as well as at lower levels in a broad population of motor neurons. (G–I) The distribution of chicken *neogenin* is a composite of the expression patterns of both mouse *Dcc* and *neogenin*. At all stages, the highest levels of *neogenin* expression is in the dorsal-most spinal cord, in dorsal neural progenitors (arrowheads, G, I) and in a population of post-mitotic dorsal neurons whose position is consistent with their being commissural neurons (arrowhead, H). Neogenin is also present at lower levels in both the ventral ventricular zone and in motor neurons (brackets, H, I). Scale bar: A, D, G: 30 µm, B, C, E, F, H, I: 40 µm.

To investigate whether chicken neogenin functionally substitutes for mouse Dcc in the developing spinal cord, we first compared the expression pattern of *neogenin* and *Dcc* in both chicken and mouse embryos in *in situ* hybridization experiments (Fig. 2). The Dcc receptor mediates the attraction of dorsal commissural axons towards netrin1 in the FP. [16], [17]. We thus examined the distribution of *Dcc* and *neogenin* during the period in which commissural neurons extend axons towards and across the FP. At embryonic (E) stage 10.5 mouse embryos, when commissural neurons are beginning to extend axons away from the dorsal midline, *neogenin* is expressed at high levels in the ventricular zone (VZ) of intermediate spinal cord (bracket, Fig. 2A). By E11.5, when many commissural axons have reached the FP, *neogenin* is now expressed throughout the ventral-most region of the VZ as well as broadly in post mitotic motor neurons (brackets, Fig. 2B, C, [16]). *Neogenin* is expressed in neural progenitors in the dorsal-most region of the VZ only at the rostral-most levels of E11.5 spinal cords (Fig. 2C). Thus, *neogenin* does not appear to be expressed in commissural neurons during the period when their axons are extending through the transverse plane of the spinal cord. In contrast, at both stages, rodent *Dcc* [16] is found at high levels in neural progenitors (arrowheads, Fig. 2D, E) and post-mitotic neurons (brackets, Fig. 2D–F) throughout the dorsal spinal cord as well as at low levels in motor neurons. The distribution of chicken *neogenin* is most consistent with being a composite of the expression patterns of rodent *Dcc* and *neogenin*. At Hamilton Hamburger (HH) stage 20 [37], which is roughly equivalent to mouse E10.5/rat E12, chicken *neogenin* is expressed at high levels in the dorsal-most spinal cord, both in the VZ and in post-mitotic commissural neurons (arrowhead, Fig. 2G). At HH stages 23 (Fig. 2H) and 26 (Fig. 2I), stages that encompass the mouse E11.5/rat E13 developmental stage, *neogenin* is present in the dorsal-most post-mitotic commissural neurons (arrowhead, Fig. 2H) as well as the ventral VZ and in motor neurons.

**Figure 3:**
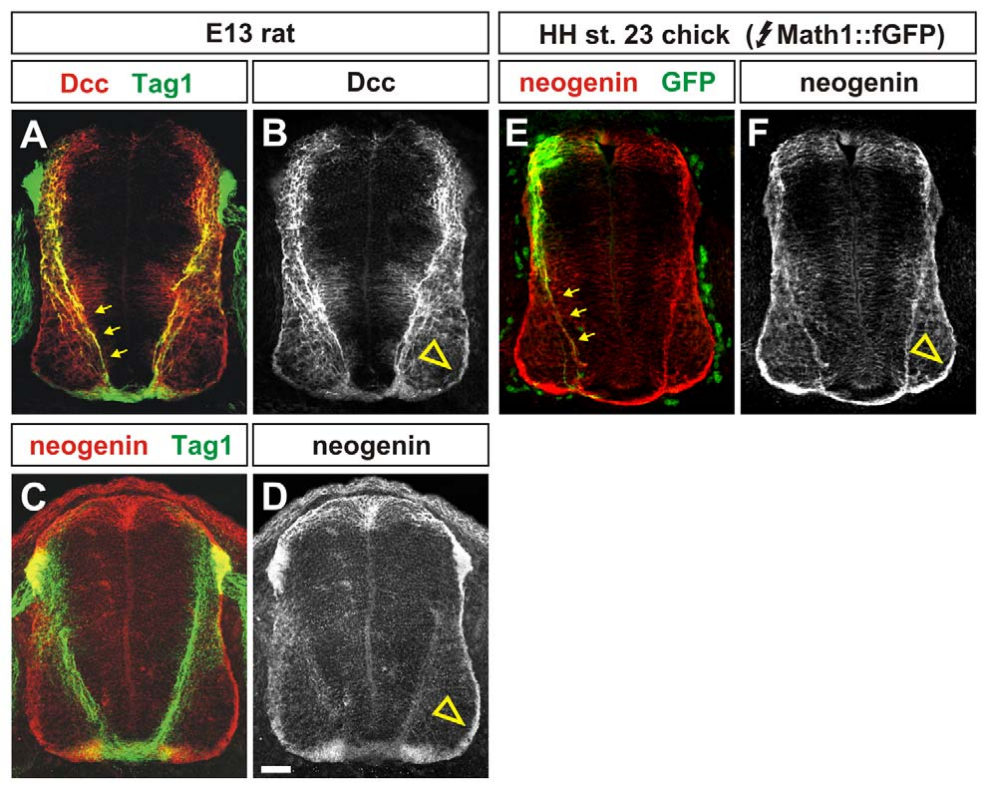
Protein distribution of neogenin and Dcc in rat and chicken embryos. (A,B) Antibodies against Dcc (red, A) label the Tag1^+^ (arrows, green, A) commissural axons dorsal spinal cord in E13 rat embryos (equivalent to E11.5 mouse embryos). Unlike Tag1, Dcc continues to be present at low levels on the post-crossing commissural axons projecting in the ventral funiculus (arrowhead, D). (C–F) Antibodies against neogenin (red, C, E) do not label the Tag1^+^ axons in E13 rat spinal cords. In contrast, in HH stage 23 chicken embryos, neogenin is present on the Math1^+^ commissural axons (arrows, green, E) targeted after *in ovo* electroporation with a Math1::farnesylated (f) GFP construct. However, in both rat and chick, neogenin is present at high levels on the post-crossing commissural axons in the ventral funiculus (arrowhead, D, F). Scale bar: A–D: 40 µm E, F: 30 µm.

We confirmed that the distribution of chicken neogenin protein is similar to the patterns of both rodent Dcc and neogenin proteins in the spinal cord (Fig. 3). In rodents, Dcc is located on Tag1^+^ commissural axons throughout the period that they are projecting through the transverse region of the spinal cord (Fig. 3A, B, [16]). Dcc remains present at low levels on the Tag1^−^ post-crossing commissural axons extending in the ventral funiculus (arrowhead, Fig. 3B, [16]). In contrast, rodent neogenin does not appear to label pre-crossing Tag1^+^ axons (Fig. 3C), but is present at high levels in the ventral funiculus (arrowhead, Fig. 3D). The distribution of chicken neogenin protein appears to be a composite of the distributions of both rodent Dcc and neogenin proteins. Chicken neogenin is present at high levels on both pre-crossing (arrows, Fig. 3E) and post-crossing (arrowhead, Fig. 3F) commissural axons. The trajectory of chicken commissural axons was revealed using a farnesylated (f) GFP reporter that our previous studies have shown labels the axonin1^+^ (the chicken homolog of Tag1, [38]) axons extending from the dorsal-most Math1^+^ commissural neurons [39].

Taken together, the results from these experiments demonstrate that the distribution of chicken neogenin has features in common with both rodent Dcc and neogenin, supporting the model that chicken neogenin functionally substitutes for Dcc in the chicken spinal cord.

### Commissural axon outgrowth is affected after the loss of either mouse Dcc or chicken neogenin

**Figure 4:**
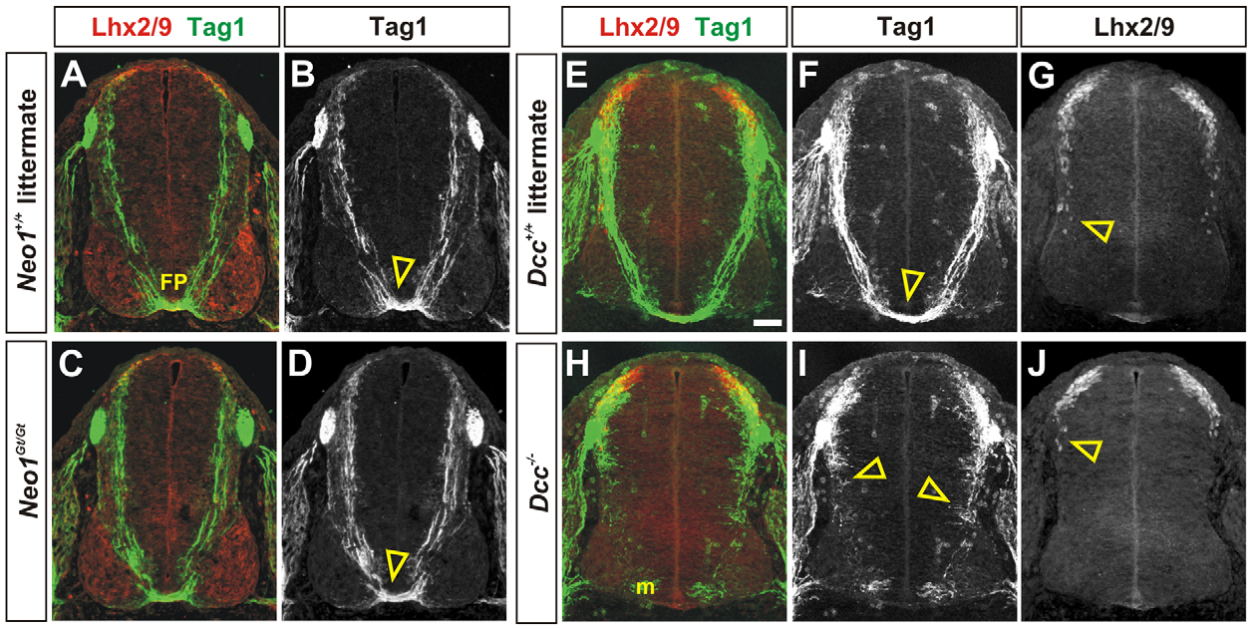
Mouse commissural axons stall in the absence of Dcc, but not neogenin. (A–J) Transverse sections taken from brachial levels of the spinal cord from E11.5 *Neo1^Gt/Gt^* (C, D) and *Dcc^−/−^* (H–I) embryos and their respective wild-type littermates (A, B, E–G) labeled with antibodies against Lhx2/9 (red, A, C, E, G, H, J) and Tag1 (green, A–F, H, I) which decorate commissural nuclei and axons, respectively. Tag1 also transiently labels motor neurons (m). (A, B, E–G) By E11.5, Tag1^+^ commissural axons in both wild-type littermate controls have extended robustly to the floor plate (FP, arrowhead, B, F). Their Lhx2/9^+^ cell bodies have also started to migrate ventrally to a deeper layer of the dorsal spinal cord (arrowhead, G). (C, D) In the *Neo1^Gt/Gt^* mutant, the extent of Tag1^+^ commissural axon outgrowth (arrowhead, D) and the migration pattern of Lhx2/9^+^ neurons is indistinguishable from control littermates. (H–J) However, in *Dcc* mutants, Tag1^+^ axons show severe defects in their extent of outgrowth: they stall in the intermediate spinal cord (arrowheads, I) with very few axons reaching the FP. There also appears to be a delay/stall in Lhx2/9^+^ cell migration (arrowhead, J) Scale bar: 40 µm.

We next compared the consequence of functionally inactivating the *Dcc* and *neogenin* genes on the commissural axon trajectory in mouse and chicken spinal cords. Although the phenotype of mouse *Dcc* loss-of-function mutations has been previously described [17], the effect of *neogenin* mutations on commissural axon guidance in the spinal cord was not known. To assess this question, a hypomorphic allele of *neogenin1*, *Neo1^Gt^*, was generated using a previously described secretory gene trap approach [40]. The trajectory of Tag1^+^ commissural axons was monitored in E11.5 mouse spinal cords. *Neo1^Gt^* mutant Tag1^+^ axons extend to the FP in an indistinguishable manner to wild-type littermate controls (arrowheads, Fig. 4B, D). In contrast, in E11.5 *Dcc^−/−^* mutant spinal cords, Tag1^+^ commissural axons extend normally away from the roof plate at the dorsal midline, but stall in the intermediate spinal cord with only a few Tag1^+^ axons projecting towards the FP (arrowheads, Fig. 4I).

**Figure 5:**
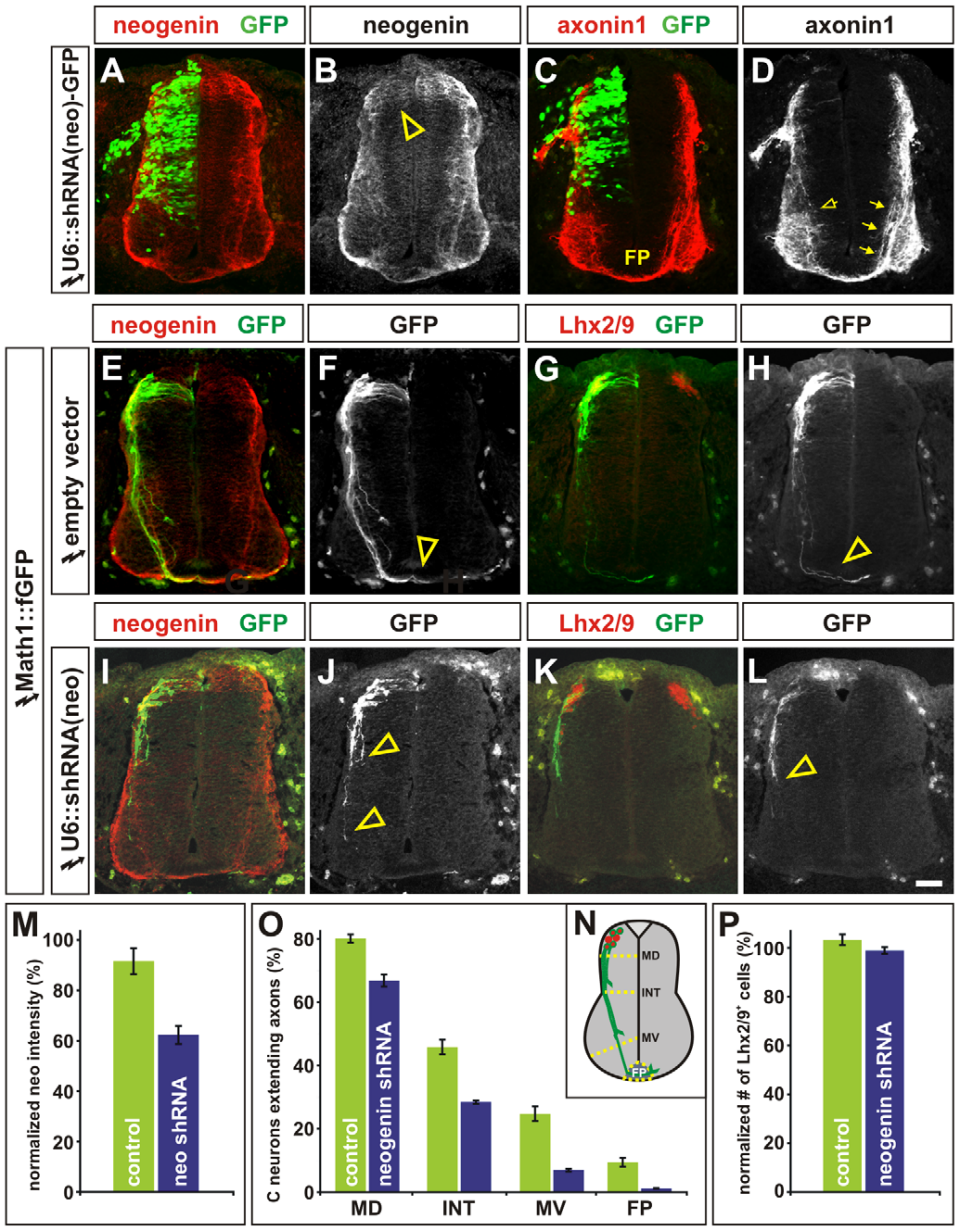
Commissural axons stall in the absence of neogenin in chicken embryos. (A–L) HH stage 14/15 chicken embryos were *in ovo* electroporated with either short hairpin micro RNAs directed against neogenin under the control of the U6 promoter (U6::shRNA(neo)-GFP, A–D, I–L) or the empty vector (E–H). This RNA interference (RNAi) vector includes GFP sequences that permit the efficacy of vector delivery to be monitored (A, C). Embryos were taken for analysis at HH stage 22. (A, B) Electroporation of the U6::shRNA(neo)-GFP construct (green) results in an observable reduction in the levels of neogenin protein (red, arrowhead, B). (C, D) The loss of neogenin (green) results in fewer axonin1^+^ axons (red) extending towards the floor plate (FP); compare thickness of the commissural axon bundle on the electroporated side (open arrow, D) to that on the non-electroporated side (arrows, D). Note that, at this stage in spinal cord development, axonin1 antibodies also label the motor axons. (E–L) To monitor the extent of axon outgrowth towards the floor plate (FP), Lhx2/9^+^ commissural neurons (red, G, H, K, L) were expressing fGFP under the control of the Math1 enhancer (Math1::fGFP, green E–L). (E–H) Math1^+^ axons project normally to the FP (arrowhead, F, H) after electroporation of the empty RNAi vector. (I–J) In contrast, the outgrowth of Math1^+^ axons is severely compromised after electroporation with U6::shRNA(neo) (arrowheads, J, H). (M) The level of neogenin protein was reduced by 38±5% (n = 12 sections from 4 embryos) on the electroporated side of the embryo compared to the non-electroporated side. This reduction in neogenin intensity is significantly different (p<2.6×10^−5^, student's t-test) from the effect of the empty vector on neogenin levels (n = 16 sections from 2 embryos). (N) The extent of commissural axon outgrowth was quantified by determining whether Math1^+^ axons had crossed one of four arbitrary lines in the spinal cord: mid-dorsal (MD), intermediate (INT), mid-ventral (MV) or the FP. (O) By HH stage 22, 80% of the control Lhx2/9^+^ neurons have extended GFP^+^ axons, of which 57% have extended into the intermediate spinal cord and 12% have reached the FP (n = 30 sections from 2 embryos). In contrast, after reducing the levels of neogenin, only 67% of the Lhx2/9^+^ neurons have extended GFP^+^ axons (p<0.013, similar to control, n = 52 sections from 4 embryos). Of these axons, 42% have projected into the intermediate spinal cord (p<0.00014, similar to control) but only 1.7% have reached the FP (p<2.9×10^−5^, similar to control), suggesting that although the experimental Math1^+^ axons largely projected to the intermediate spinal cord, they did not proceed further. (P) This defect did not result from a loss of Lhx2/9^+^ neurons. The number of Lhx2/9^+^ neurons on the U6::shRNA(neo)-GFP electroporated side was statistically indistinguishable from either the non-electroporated side (p>0.65, similar to control) or control electroporations (p>0.07, similar to control. Scale bar: 30 µm.

We then used an RNA interference (RNAi) approach to assess the consequence of reducing the level of neogenin protein in the chicken spinal cord. RNAi knockdown was achieved by inserting two micro (mi) RNA30-like hairpins directed against the *neogenin* mRNA into a vector designed for optimal gene silencing in chick embryos [41]. This vector uses a chick U6 enhancer to drive expression of the miRNAs, which is more effective in chick embryos than enhancers of mammalian origin [41]. The RNAi vector also contains an GFP sequence to permit plasmid delivery to be monitored (Fig. 5A, C). This construct was *in ovo*-electroporated into HH stage 14/15 chicken embryos, just before commissural axon extension begins, and the resulting embryos were harvested at HH stage 22, when commissural axons are in the process of projecting to the FP. *Neogenin* RNAi knockdown was successful; the level of neogenin protein was reduced by an average of 40% (arrowhead, Fig. 5B, M). Moreover, neogenin knockdown resulted in far fewer axonin1^+^ [38] axons projecting to the FP on the electroporated side (open arrow, Fig. 5D) compared to the non-electroporated side (arrows, Fig. 5D).

To quantify this phenotype without any confounding signal from the motor axons, we examined the extent of axon outgrowth from the Math1^+^ commissural neurons, by co-electroporating a Math1::fGFP reporter [39] in addition to the *neogenin* RNAi vector. The Math1 enhancer has been shown to direct the expression of genes specifically to post-mitotic Lhx2/9^+^ commissural neurons [39], [42]. In control experiments using the empty vector, GFP^+^ commissural neurons projected many axons normally towards and across the FP by HH stage 22 (arrowheads, Fig. 5E–H). In contrast, after neogenin knockdown, the trajectory of Math1^+^ commissural axons was severely affected with very few axons reaching the FP (arrowheads, Fig. 5I–L).

We quantified the extent of axon outgrowth by determining whether the Math1^+^ axons had crossed one of four arbitrarily chosen boundaries in the spinal cord: mid-dorsal (MD), intermediate (INT) or mid-ventral (MV) spinal cord or the FP (Fig. 5N, [39], [43]). In control embryos, 57% of Lhx2/9^+^ neurons had extended GFP^+^ axons to the INT line and 12% had reached the FP by HH stage 22 (Fig. 5O). However, at the same stage in the neogenin knockdown embryos, 43% of Lhx2/9^+^ neurons had extended GFP^+^ axons to the INT line but only 2% had reached to the FP (Fig. 5O). Thus, following neogenin knockdown, many of the Math1^+^ commissural axons stall above the FP, a defect in axonal outgrowth similar to that observed in the mouse *Dcc^−/−^* mutants. This defect did not result from an alteration in commissural neural fate, similar numbers of Lhx2/9^+^ (Lh2a/b) neurons [44] were observed on the electroporated and non-electroporated sides in both control and experimental conditions (Fig. 5P).

Taken together, the analysis of neogenin knockdown in chicken spinal cords reveals a striking similarity with the *Dcc* mutant phenotype in mice. Commissural axons show a profound axon extension phenotype: they stall in the ventral spinal cord, consistent with their no longer being directed towards the netrin1 attractant at the FP [17], [36].

## Discussion

Dcc is a critical factor in the formation of neural circuits throughout the nervous system. Thus, it is somewhat surprising that a Dcc homolog has yet to be identified in the chick genome. Our searches also failed to find a Dcc homolog in both the zebra finch and turkey genomes, suggesting that the loss of the *Dcc* gene may be common to avian genomes. It is unlikely that this observation results from Dcc-netrin signaling having no role in the formation of avian neural circuits given that the role of netrin as an axon guidance signal is evolutionary highly conserved [10]. Moreover the chemoattractive activity of netrin1 for commissural axons was first identified in chicken embryos [45] suggesting that there must be a receptor on commissural axons that mediates their trajectory towards the FP. Here, we suggest that this role is assumed by chicken neogenin: chicken neogenin is present in a similar distribution to both rodent Dcc and neogenin and the loss of neogenin by RNA interference phenocopies the effect of a *Dcc* loss-of-function mutation in mice.

However, it remains a possibility that the chick Dcc homolog is located in the 5% of the chicken genome that remains unsequenced. We think this possibility is unlikely. Previous attempts to amplify a chick Dcc homolog from brain mRNA libraries have failed [21]. Moreover, by comparing the synteny of the mouse chromosome around the *Dcc* locus to that of the chicken genome, we have found that the equivalent chicken locus is in the region of a previously described translocation between chicken chromosome 2 and Z [1]. It is possible that the *Dcc* gene was lost during this rearrangement.

### The expression patterns and loss of function phenotypes of rodent Dcc and chicken neogenin are strikingly similar in the spinal cord

The expression pattern of chicken *neogenin* is consistent with it being a composite of the expression patterns of both rodent *neogenin* and *Dcc* suggesting that chicken neogenin may perform the roles of both rodent Dcc and neogenin during development. However, further investigation of whether chicken neogenin also performs the analogous role of rodent neogenin awaits a deeper understanding of the function of neogenin in the developing rodent spinal cord.

The effect of reducing either chicken neogenin or mouse Dcc protein levels on commissural axon outgrowth is strikingly similar: the axons stall above the ventral midline. The *Dcc* mutant spinal cords also show a previously unreported Lhx2/9^+^ cell migration phenotype, the cell bodies of the commissural neurons appear delayed in their ability to migrate into the deeper layers of intermediate spinal cord. This phenotype may be a secondary consequence of the commissural axons having stalled, the neurons are thereby unable to migrate very far ventrally along the paths of their own axons. Chicken commissural neuron migration starts at a later stage than HH stage 22 i.e. the process is normally delayed with respect to rodents. We saw no defect in migration of commissural neurons after knockdown with neogenin shRNAs (data not shown). However this experiment required us to incubate the embryos to HH stage 27, when we also observed significantly diminished expression of the neogenin RNAi vector. Thus, the RNAi approach may not be sufficiently long-lived to satisfactorily address whether chicken neogenin also regulates the ventral migration of commissural neurons.

### Functional equivalence of Dcc and neogenin

Finally, it remains unresolved whether chick neogenin is the functional equivalent of rodent neogenin or whether it has a hybrid activity, combining the activities of both Dcc and neogenin. Neogenin has been shown to bind various netrins as well or better than Dcc [16], [28] in addition to the RGM ligand [26]. Structure-function analysis of both neogenin and Dcc has suggested that the FN type III repeats mediate ligand binding. Netrin has been shown to bind to either the FNIII-4 [5] or FNIII-5 domains [4], [6]. In contrast, RGMc has been shown to bind to the FNIII-5 and 6 repeats [46]. It is thus interesting to note that although the amino acid sequence of FNIII-4 and 6 is 100% identical between the Neo or Dcc orthologs, there are two non-conserved amino acid substitutions in the FNIII-5 domain of chicken neogenin (highlighted, Fig. 1B) that are more similar to human/mouse Dcc than human/mouse neogenin1 (note that the overall level of similarity within FNIII-5 domain is still highest between the orthologs). Future rescue experiments and/or site-directed mutagenesis will resolve whether these residues are key to the ability of chick neogenin to assume the functions of rodent Dcc, perhaps by altering the specificity of ligand binding.

In summary, we have shown that chicken neogenin appears to functionally substitute for Dcc in the developing spinal cord. Future studies will also determine whether neogenin substitutes for the function of Dcc in other developing systems in the chicken embryo, such as the visual system where Dcc has a key role guiding retinal ganglion axons into the optic nerve [20].

## Materials and Methods

### Ethics statement

All animal work was performed strictly in accordance with both University of Southern California Institutional Animal Care and Use Committee (IACUC) and US National Institutes of Health laboratory animal care guidelines. All efforts were made to minimize the suffering of animals. The animal protocol (#10948) for these studies was approved by IACUC on 21^st^ October 2009. The institutional animal assurance welfare number is A3518-01.

### Immununohistochemistry and *in situ* hybridization

Antibody staining and *in situ* hybridization histochemistry was performed on either cryosectioned, dissociated neurons or whole mount tissues as previously described [47]. Fluorescence and DIC images were collected on a Zeiss LSM510 confocal and Axiovert 200 M and Axioplan 2 microscopes. Images were processed using Adobe Photoshop CS2 and CS4.

The antibodies against the following proteins were used: **mouse:** Tag1 at 1∶6 (4D7, [12], GFP at 1∶2000 (3E6, Invitrogen), Dcc at 1∶1000 (Calbiochem) **rabbit:** neogenin at 1∶500 (Santa Cruz Biotechnology) panLh2 (Lhx2/9) at 1∶1000 (L1, [11], axonin1 at 1∶10,000 [38]. Cy3-, Cy5- or FITC-coupled secondary antibodies were obtained from Jackson Immunoresearch.

For the *in situ* hybridization experiments, the Primer3 online program (http://frodo.wi.mit.edu/) was used to select optimal primer sequences that would result in a PCR product size of 300–400 base pairs (bp) and an annealing temperature of 60°C. The following primers were used to generate anti sense *in situ* probes against 1) mouse *Dcc* (accession number, NM_007831, Genbank), forward: 5′-GAG TAT TTA GGT GAC ACT ATA G-3′, reverse: 5′-GAC ACA ATC AGC AGC AGG AA-3′, SP6 site, 2) mouse *neogenin1* (BC_054540), forward: 5′- GAGATT AACCCTCACTAAAGGGA-3′, reverse: 5′-ATT GTG TTT GGA ATG CAC CA-3′, T3 site, 3) chick *neogenin* (XM_413704), forward: 5′-GAG TAA TAC GAC TCA CTA TAG GG-3′, reverse: 5′-ATG CGA ATG TCC AAC ACT GA-3′, T7 site. In all cases, the indicated RNA polymerase binding sequences were added 5′ to the anti-sense DNA sequence. The target sequence was amplified from either E10.5 mouse or HH stage 18 chicken spinal cord cDNAs by PCR. Qiaquick (Qiagen) purified products were used in an *in vitro* transcription reaction using the Roche DIG RNA labeling kit. *In situ* hybridization experiments were performed using standard techniques [48].

### Generation and analysis of mutant mice

Mice carrying a secretory gene-trap vector insertion into intron 7 of the *neogenin* gene were developed using a previously described embryonic stem (ES) cell line [40]. The ES cell line KST265 was obtained from BayGenomics and chimeric mice were generated by tetraploid aggregation. Characterization of the hypomorphic nature of a mouse line developed using this ES cell line has been previously reported [49].

*Dcc* and *neogenin* mutant embryos were genotyped by PCR either as previously described [17] or using the following primers, Neo-1: 5′-CAA CCT GGG TCA TAG CAA TGG-3′, Neo-2: 5′-GTG TGA ACA AAG AAG CAG AAA GGC-3′, Neo-3: 5′-TAA ATG ACG ACA CAG CAG GCC-3′ at an annealing temperature of 55°C. This *neogenin* PCR reaction generates a 917 bp mutant band and a 704 bp wild-type band.

### Generation and analysis of short hairpin RNAs

To specifically reduce the expression of *neogenin* by *in ovo* electroporation of chicken embryos, we designed target sequences using the design tool at www.genscript.com/ssl-bin/app/rnai and the *Gallus gallus neogenin* nucleotide sequence, accession number XM_413704 (Genbank). These target sequences were then incorporated into micro RNA30-like hairpin structures in a derivative of the pRFPRNAiC vector [41], in which GFP was substituted for RFP. This plasmid is referred to as U6::shRNA-GFP. A version of this vector was also generated that did not express GFP, U6::shRNA.

Target sequence for first hairpin oligos, designed from *neogenin* nucleotides, 3262–3282: Forward: 5′-GAG AGG TGC TGC TGA GCG **C** TG GAT AGC AAC ATG CTT CTT TAG TGA AGC CA CAG ATG TA-3′. Reverse: 5′-ATT CAC CAC CAC TAG GCA TTG GAT AGC AAC ATG CTT CTT TAC ATC TGT GGC TTC ACT-3′. Target sequence for second hairpin oligos, designed from *neogenin* nucleotides, 135–155: Forward: 5′-CTG GTT CCT CCG TGA GCG **C** TA TTG TGA AAC TCC TCC GAA TAG TGA AGC CAC AGA TGT A-3′. Reverse: 5′-CCT GAA GAC CAG TAG GCA TTA TTG TGA AAC TCC TCC GAA TAC ATC TGTGGCTTCACT. Note: in both cases, the underlined region is the targeted chicken sequence and the bold reside is purposely mismatched.

These oligos were cloned by PCR using Expand High Fidelity Plus Taq polymerase (Roche) with the following flanking primers to generate the DNA fragment for the first and second hairpin, respectively. 1^st^ hairpin primer 5′: 5′-GGC GGG GCT AGC TGG AGA AGA TGC CTT CCG GAG AGG TGC TGC TGA GCG-3′; 1^st^ hairpin primer 3′: 5′-GGG TGG ACG CGT AAG AGG GGA AGA AAG CTT CTA ACC CCG CTA TTA CCA CCA CTA GGC A-3′; 2^nd^ hairpin primer 5′: 5′-GGC GGG ACG CGT GCT GTG AAG ATC CGA AGA TGC CTT GCG CTG GTT CCT CCG TGA GCG-3′; 2^nd^ hairpin primer 3′: 5′-CGC CGC GCA TGC ACC AAG CAG AGC AGC CTG AAG ACC AGT AGG CA-3′. These PCR products were cloned into U6::shRNA-GFP using the Nhe I and Mlu I (first hairpin) and Mlu I and Sph I (second hairpin) restriction sites. Rescue experiments were not attempted given the likelihood of confounding gain of function phenotypes arising from the overexpression of Dcc in chicken spinal cord.
